# The Journey From Nonimmersive to Immersive Multiuser Applications in Mental Health Care: Systematic Review

**DOI:** 10.2196/60441

**Published:** 2024-11-07

**Authors:** Iveta Fajnerova, Lukáš Hejtmánek, Michal Sedlák, Markéta Jablonská, Anna Francová, Pavla Stopková

**Affiliations:** 1 Research Center for Virtual Reality in Mental Health and Neuroscience National Institute of Mental Health Klecany Czech Republic; 2 Third Faculty of Medicine Charles University Prague Czech Republic; 3 Faculty of Humanities Charles University Prague Czech Republic

**Keywords:** digital health, mental health care, clinical interventions, multiuser, immersive, virtual reality, VR, app, mental health, online tools, synthesis, mobile phone, PRISMA

## Abstract

**Background:**

Over the past 25 years, the development of multiuser applications has seen considerable advancements and challenges. The technological development in this field has emerged from simple chat rooms through videoconferencing tools to the creation of complex, interactive, and often multisensory virtual worlds. These multiuser technologies have gradually found their way into mental health care, where they are used in both dyadic counseling and group interventions. However, some limitations in hardware capabilities, user experience designs, and scalability may have hindered the effectiveness of these applications.

**Objective:**

This systematic review aims at summarizing the progress made and the potential future directions in this field while evaluating various factors and perspectives relevant to remote multiuser interventions.

**Methods:**

The systematic review was performed based on a Web of Science and PubMed database search covering articles in English, published from January 1999 to March 2024, related to multiuser mental health interventions. Several inclusion and exclusion criteria were determined before and during the records screening process, which was performed in several steps.

**Results:**

We identified 49 records exploring multiuser applications in mental health care, ranging from text-based interventions to interventions set in fully immersive environments. The number of publications exploring this topic has been growing since 2015, with a large increase during the COVID-19 pandemic. Most digital interventions were delivered in the form of videoconferencing, with only a few implementing immersive environments. The studies used professional or peer-supported group interventions or a combination of both approaches. The research studies targeted diverse groups and topics, from nursing mothers to psychiatric disorders or various minority groups. Most group sessions occurred weekly, or in the case of the peer-support groups, often with a flexible schedule.

**Conclusions:**

We identified many benefits to multiuser digital interventions for mental health care. These approaches provide distributed, always available, and affordable peer support that can be used to deliver necessary help to people living outside of areas where in-person interventions are easily available. While immersive virtual environments have become a common tool in many areas of psychiatric care, such as exposure therapy, our results suggest that this technology in multiuser settings is still in its early stages. Most identified studies investigated mainstream technologies, such as videoconferencing or text-based support, substituting the immersive experience for convenience and ease of use. While many studies discuss useful features of virtual environments in group interventions, such as anonymity or stronger engagement with the group, we discuss persisting issues with these technologies, which currently prevent their full adoption.

## Introduction

### Background

Thousands of studies are exploring self-care applications, which deliver immediate on-demand psychological help to people otherwise waiting on in-person therapy [1,2]. There are a multitude of research projects aimed at exploring the efficiency of online tools or virtual environments for addressing anxiety [2] or affective disorders [3]. However, most of the current investigation into technologies and mental health care is focused on single-user experiences [4-6].

One of the determining factors of effective psychotherapy is establishing a functional relationship between the therapist and the client [7]. In the case of group interventions, the establishment of mutual communication between clients in the group plays a crucial role [8]. However, while technological advances have modernized, simplified, and increased the availability of therapeutic interventions to people who might have had difficulties accessing them in the past, these tools are largely solitary. Meanwhile, multiuser tools and virtual environments could provide useful intervention programs for a therapeutic dyad or a whole group (3 or more participants [9,10]).

There are a number of social groups meeting and supporting each other in various areas related to mental disorders and mental health, such as postnatal depression, addiction, or suicidal tendencies [11-14]. People search for peer support not only on social media but also in different virtual environments, where they form dedicated groups. Such support groups have appeared in many popular video games, such as World of Warcraft (Blizzard Entertainment), Second Life (Linden Research, Inc), or Minecraft (Mojang studios) [15,16] or recently in virtual reality (VR) enabled social applications, especially VRChat (VRChat, Inc) [17-20]. Currently, we can already recognize a whole range of commercial VR applications designed as platforms for mutual communication and interaction in virtual environments, often referred to as the *metaverse* [21], such as Meta Horizon workrooms (Meta Inc) [22], Rec room (Meta Inc) [23], or Glue (Meta Inc) [24].

### This Review

This review aims to focus on the applications and procedures of mental health care interventions that use such multiuser platforms, where professionals or peers work with clients side by side and which use technology as a means to connect, not to substitute the human contact (ie, self-care apps or artificial intelligence (AI) chatbots [25,26]).

We set off to answer the following questions: (1) what has changed in the field of remote group therapy in the past 25 years, (2) what technologies have been tested and deemed functional, (3) which groups seem to benefit most from such interventions, and (4) what new platforms, including immersive VR, have to offer with regard to future mental health support.

### Group Therapy and Its Role in Mental Health Care

Group psychotherapy or group therapy is a form of psychotherapy that involves treating a small number of clients under the supervision of one or more therapists [27]. A broader concept of group therapy refers also to support groups for people with a variety of mental health conditions usually led by peers aimed at psychoeducation and skills training [28].

Group psychotherapy is based not only on the interaction of therapists with a group of patients but also on the interactions between patients or clients themselves [29]. In the original concept of psychodynamic and interpersonal group therapy, the group dynamics, that is, relationships and interactions between members and the therapist, are used for therapeutic purposes [29,30]. The group context and group process are considered the main mechanisms of change by developing, exploring, and examining interpersonal relationships within the group.

Group psychotherapy offers several advantages over an individual therapy format [31]. First, more patients can reach the treatment at the same time, at a reasonable cost, and one therapist may interact with several patients at the same time. This is highly important given the fact that in some countries, only a small number of psychiatric patients actually receive adequate psychological care [32].

Second, the experience of being a part of the group itself, sharing personal experiences, and meeting other people with similar mental health problems can have therapeutic effects [33], as is the case of self-care or support groups. Other treatment factors specific to group therapy that emerge from the interpersonal setting include feeling connected to the world and being respected and valued by others [34]. Other specific treatment factors were proposed, including vicarious and interpersonal learning, social support, experiencing universality, altruism, fostering hope, and a sense of belonging and relatedness [35]. Group therapy may therefore provide a source of corrective relational experiences [36,37]. Some studies have shown better ratings of group therapeutic factors during ongoing treatment, as well as a relationship to treatment outcome for patients with anxiety disorders, although systematic evaluation of these factors is inadequate [38-40]. Therapeutic factors and group processes in online groups appear, thus far, to be the same as those in traditional group interventions [41,42].

Needless to say, there are disadvantages to group therapy. Participants must be willing to share their personal experiences and issues. Some patients consider this format so challenging that it can prevent their engagement in the group, which becomes an obstacle to successful treatment [43]. The group setting also provides less time and opportunities to deal with individual topics, and some patients may feel overlooked [44]. Organization of group meetings is more difficult and managing the therapeutic group requires additional skills and specific training from the therapist compared with individual therapy [43].

In the context of cognitive behavioral therapy (CBT), the effectiveness of group therapy is well-supported [45]. The results of a meta-analytic review indicated that anxiety prevention group programs can be a promising strategy for reducing the incidence rates of anxiety disorders [46]. Researchers have not found substantial differences between group and individual CBT for treatment of anxiety disorders in adults [47,48], as well as in youth [49,50]. Group CBT was also superior to waitlist control groups or produced equivalent results when compared with other active treatments, that is, individual psychotherapy and pharmacotherapy [47,51,52].

### Online Communication in Health Care: From Chat Rooms to Virtual Worlds

Digital tools have considerably affected how people manage their mental health. The accessibility of online social support has been gradually rising due to free social media and communication platforms [53,54]. People look on the web for information and knowledge about their condition, but they also seek guidance and support from peers and professionals [53,55]. Over the past 25 years, there has been a remarkable evolution in how people connect and communicate in online spaces in general. The rapid technological development is clearly visible in the transition from text-based chat rooms to videoconferencing, which represents a substantial shift in the way we interact with others on the web. The recent emergence of “virtual worlds” and immersive virtual devices provides another crucial milestone in digital interactions. So how have these technologies been used in mental health care over the years?

In the late 1990s and early 2000s, online communication primarily revolved around text–based chat rooms. These platforms allowed users to engage in real time conversations with others who shared similar interests or demographics. However, interactions in chat rooms were limited to text, and there was minimal visual or auditory engagement. This approach has been mostly used in the form of peer-support groups and peer-to-peer communities, mostly addressing depression and social support, in general [56]. Even now, chat rooms are extensively used in various forms. Social networking sites have gained massive user bases, presenting an opportunity to deliver internet-based mental health interventions to many people. A review covering the application of social networking sites in mental health care [57] pointed out that such an approach, mostly aimed at mental health literacy or specific symptoms (depression), shows high acceptability and engagement. Despite promising results, there is a lack of high-quality evidence supporting its effectiveness.

Voice calls became more prevalent in online spaces during the mid-2000s. A voice added a new dimension to online interactions, enhancing the sense of presence and immediacy and allowing for certain nonverbal cues (eg, voice intonation, speech speed, and pauses). An even bigger improvement came in the late 2000s when videoconferencing (eg, Skype, Zoom, and Microsoft Teams) became available. Videoconferencing provides a more immersive experience, by allowing participants to see each other’s facial expressions, gestures, and partially, their body language, all in real time. Videoconferencing is also simple to use, and therefore, it has become a popular platform in remote health care group interactions. A systematic review of home-based support groups delivered via videoconferencing showed that this approach is feasible, as it can replicate group processes, such as bonding or cohesiveness with outcomes similar to in-person groups [58]. It demonstrated the ability to engage with people with similar problems; improve accessibility to peer groups; and development of health-related knowledge, skills, and insights provided during the intervention.

Another essential advancement came with the development of “virtual worlds” or virtual environments. These were originally designed as games (eg, Second Life, World of Warcraft, and Minecraft), but at its core, they can be seen as social platforms as well. Besides the often implemented voice and chat-based messaging, these worlds also allow for interpersonal interactions, such as approaching somebody or avoiding them, exchanging items, or emoting. Although people usually start playing for fun, these games have a lot of downtime or routine work that does not require constant attention, which invites socializing with other players. For these reasons, virtual worlds provide a well-featured place where support groups can organically appear [16].

In recent years, a range of extended reality technologies have gained traction, offering even more immersive and interactive experiences in virtual worlds. These immersive technologies include VR, augmented reality (AR), and mixed reality (MR). AR and MR lenses like Pokémon GO and Snapchat overlay digital content onto the real world, blending virtual and physical experiences. Immersive VR headsets (head-mounted displays [HMDs]) like HTC Vive and Meta (Oculus) Quest allow users to fully enter virtual worlds and interact with the environment and other people as well. Virtual worlds (eg, metaverse) and immersive headsets offer novel opportunities for delivering group–based mental health interventions. These technologies can simulate real-life social interactions, provide engaging and interactive experiences, and enhance the sense of presence and connection among participants [59,60].

The importance of internet-based multiuser tools and platforms for health care has been enhanced during the COVID-19 pandemic (beginning to emerge worldwide in 2020), which has exacerbated social isolation and mental health challenges [61], particularly among marginalized communities (eg, older mothers of newborns, cultural minorities, and people with various psychiatric disorders). These groups often face unique challenges that contribute to feelings of isolation, including limited access to resources, societal stigma, and changes in social support networks. Therefore, we assume that the occurrence of tools supporting social communication in mental health care during COVID-19 has increased rapidly during the pandemic [62,63], as well as the number of extended reality applications focused on mental health in general.

### Objectives of the Systematic Review

This systematic review aimed to investigate the use of online multiuser interventions to support mental health that facilitate communication in dyadic and group interventions in the last 25 years. The main objective was to systematically explore and interpret evidence about these types of interventions, specifically about methods and systems (multiuser technologies) used. This reflects the absence of review studies that look into this phenomenon comprehensively and not only with respect to the selected technology (eg, chat rooms). The second objective was to evaluate acceptability, suitability, and safety of multiuser technologies, and identify potential gaps and opportunities for future research. Specifically, this review aimed to identify potential benefits and disadvantages of the available multiuser technologies, especially with regard to their use in therapeutic or peer-supported group interventions. Furthermore, our aim was to understand whether the potential of immersive technologies has manifested itself in an increase in studies using VR or AR multiuser interventions, and what barriers might prevent it.

## Methods

### Review Guidelines

This systematic review includes primary sources related to the use of multiuser interventions in the context of mental health care. It follows the principles of the updated PRISMA (Preferred Reporting Items for Systematic Reviews and Meta-Analyses) 2020 guideline [64,65].

### Inclusion and Exclusion Criteria

Several inclusion and exclusion criteria were determined before and during the records screening process. The database search covered articles in English, published from January 1999 to March 2024, related to multiuser mental health interventions. For the identification of records, the inclusion criteria were established as follows: (1) the record should be related to mental health care, (2) the intervention should be conducted in 2 or more people, and (3) the intervention itself should be administered or mediated via digital technology. Opinion papers, research protocols, review papers, and papers without original research were excluded.

### Search Strategy

Two academic database sources, Web of Science and PubMed, were used for the systematic review search using the search queries listed in Table 1.

Both queries performed the search in the title, abstract, and keywords of the database records. Specifying keywords in the queries for the term “multiuser” was crucial to incorporate studies that used inconsistent terminology. By adding a wide range of alternative keywords, such as, “multi patient,” “dyadic,” or “group therapy,” the number of results from the databases dramatically increased. The export from the databases was conducted on April 09, 2024. All records were screened based on the predefined inclusion criteria.

**Table 1:** Database search queries for records identification. YP: Year published; TS: Topic.

Database	Search queries
Web of Science	PY=(1999-2024) AND TS=((virtual OR digital OR app OR “computer program”) AND (mental OR psychological OR psychiatric OR depress* OR anxiety) AND (therapy OR teletherapy OR psychotherapy OR intervention OR treatment) AND (multi-user OR multiuser OR “multi user” OR multi-patient OR multipatient OR “multi patient” OR multi-participant OR multiparticipant OR “multi participant” OR multi-respondent OR multirespondent OR “multi respondent” OR multi-proband OR multiproband OR “multi proband” OR dyadic OR triadic OR collaborative OR cooperative OR “group therap*” OR “group teletherap*” OR “group intervention*” OR “group treatment*” OR “group support*” OR “therapy group*” OR “teletherapy group*” OR “intervention group*” OR “treatment group*” OR “support group*”))
PubMed	(1999:2024[dp]) AND (virtual[Title/Abstract] OR digital[Title/Abstract] OR app[Title/Abstract] OR “computer program”[Title/Abstract]) AND (mental[Title/Abstract] OR psychological[Title/Abstract] OR psychiatric[Title/Abstract] OR depress*[Title/Abstract] OR anxiety[Title/Abstract]) AND (therapy[Title/Abstract] OR teletherapy[Title/Abstract] OR psychotherapy[Title/Abstract] OR intervention[Title/Abstract] OR treatment[Title/Abstract]) AND (multi-user[Title/Abstract] OR multiuser[Title/Abstract] OR “multi user”[Title/Abstract] OR multi-patient[Title/Abstract] OR multipatient[Title/Abstract] OR “multi patient”[Title/Abstract] OR multi-participant[Title/Abstract] OR multiparticipant[Title/Abstract] OR “multi participant”[Title/Abstract] OR multi-respondent[Title/Abstract] OR multirespondent[Title/Abstract] OR “multi respondent”[Title/Abstract] OR multi-proband[Title/Abstract] OR multiproband[Title/Abstract] OR “multi proband”[Title/Abstract] OR dyadic[Title/Abstract] OR triadic[Title/Abstract] OR collaborative[Title/Abstract] OR cooperative[Title/Abstract] OR “group therap*”[Title/Abstract] OR “group teletherap*”[Title/Abstract] OR “group intervention*”[Title/Abstract] OR “group treatment*”[Title/Abstract] OR “group support*”[Title/Abstract] OR “therapy group*”[Title/Abstract] OR “teletherapy group*”[Title/Abstract] OR “intervention group*”[Title/Abstract] OR “treatment group*”[Title/Abstract] OR “support group*”[Title/Abstract])

### Data Selection Process and Records Eligibility

Out of 2679 records obtained from the databases, 1540 (57.48%) were from Web of Science and 1139 (42.52%) were from PubMed. These were processed by automation tools; 987 (36.84%) were identified as duplicates and 56 (2.09%) were marked as ineligible as they were incomplete or did not fit the publication type criteria (eg, poster abstracts). This resulted in 1636 (61.07%) records being moved from the identification phase to the screening phase.

The screening phase consisted of 3 manually conducted screenings with the help of a self-hosted NocoDB database viewer [66]. The records were assessed by 5 reviewers consisting of experienced scientific researchers of our team. In screenings 1 and 2, the reviewers read through the abstracts of all records and tagged them with predefined categories based on the inclusion and exclusion criteria. Screening 3 consisted of reading and assessing full-texts of the records.

Screening 1 started with 1636 (61.07%) records, and each record was assessed by a 1 reviewer (from 5 reviewers in total). The listed reasons for excluding the record were: (1) if it was not aimed at mental health (eg, focused on the use of technologies in physical therapy), (2) it did not include any multiuser experiences (eg, use of self-care apps), (3) it was not delivered through digital mediums (eg, digital medium used for scheduling sessions or data recording, not for therapy), (4) it was discovered to be a duplicate, (5) it was a protocol or an opinion without data or (6) other, unspecified reasons. This process resulted in the exclusion of 1310 (48.9%) records.

Screening 2 started with 326 (12.17%) records and each record was assessed by multiple reviewers to achieve objectivity. Every record that was tagged by at least 3 reviewers as not matching inclusion criteria was excluded. The exclusion criteria were similar to those in screening 1. However, to limit the number of records, we decided to exclude records that dominantly focused on mental health care using internet-based tools in response to the COVID-19 pandemic in healthy populations. We also excluded records focusing solely on secondary caregivers (eg, peer support groups for parents of psychiatric patients), and interventions using AI chatbots. This resulted in 171 (6.38%) records being excluded.

There was an intermediate step in the screening process. It started with 155 (5.79%) records and consisted of filtering out the records that were tagged as being reviews during screening 1 and 2. This resulted in the exclusion of 83 (3.1%) records.

Screening 3 started with 72 (2.69%) records. In this phase, every author focused on extracting relevant information through independent full-text reviews. During this process, another 23 (0.86%) records were excluded, the most common reason being that the intervention was not in fact multiuser or that the record only described the opinions or insights of the authors.

In total, 49 (1.83%) records were considered eligible and included in our systematic review. The full-texts of these records were read and summarized, each by a single researcher, and information about procedures and study designs, means of intervention administration (technology, hardware), target groups, sample sizes, outcome measures, and other parameters were extracted.

## Results

### Standardization of Terminology

The performed systematic review had to overcome considerable complications related to unclearly defined or not established terminology regarding multiuser online tools. Studies using these technologies adopted very broad terminology based on the context of internet-based platforms used, often referring to virtual environments ranging from simple web-based forums to virtual worlds. We decided to distinguish these technologies as chat rooms, voice calls, videoconferences, and virtual worlds. The virtual worlds include both low-immersive desktop use to immersive visualization using VR headsets. A much bigger challenge was the search for applications that are designed for use by multiple users, allowing communication and interaction. Here, the terminology was quite diverse, with the term multiuser appearing only in a few cases. Some of the applications refer to communication or collective applications, but most of the records simply referred to group interventions or therapies, which alone made the search strategy very difficult, as the term “group” is often used in connection with the research method (experimental, therapeutic, or control group). Thus, the search required a very laborious elimination of all false-positively selected articles that did not address any group activities. This inconsistency in terminology regarding the term “multiuser” creates a potential negative selection bias when searching for records in the scientific body of knowledge. Despite the elaborated query incorporating many possible variations of the terms used for multiuser technologies, some records could be potentially missed if the research teams did not use all the terms included in our search query.

### Multiuser Interventions in Mental Health Care

**Figure 1:**
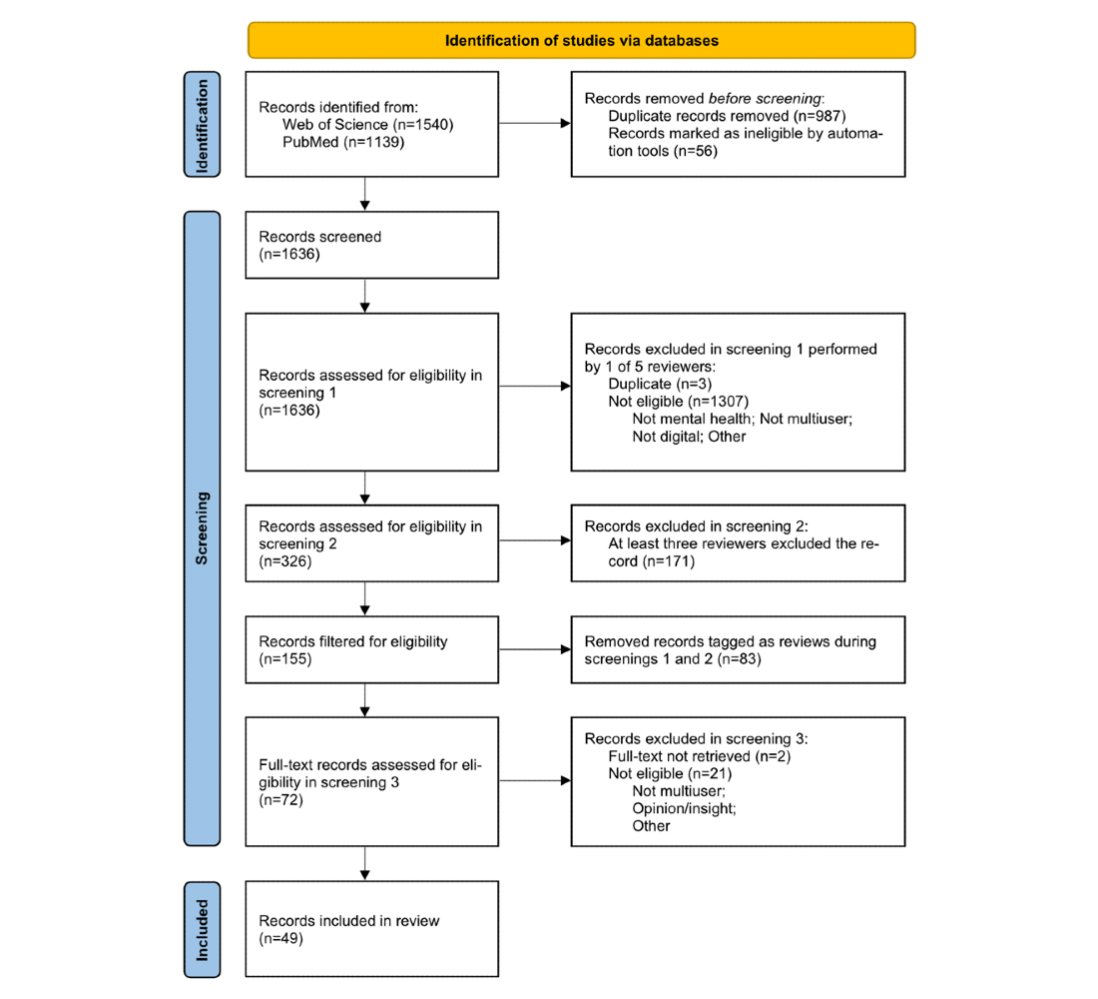
PRISMA (Preferred Reporting Items for Systematic Reviews and Meta-Analyses) 2020 flow diagram for the overview of the selection process.

An overview of the records selection process is documented in the PRISMA 2020 flow diagram (Figure 1). The systematic search led to 49 (1.83%) records that present studies aimed at dyadic and group interventions. Even though we covered 25 years of progress, it was apparent that most of the published works appeared after 2015 (only 2 records appeared before). This suggests that even though chat rooms and videoconferencing were available for more than a decade sooner, they did not find their way to clinical care as fast as could be expected.

As mentioned before, we were interested both in dyadic interventions, allowing communication between the patient (client) and the professional (expert), or peer and group interventions, allowing interaction of 3 or more participants. Out of 49 studies, our search identified 10 (20%) studies that applied purely dyadic interventions, 36 (73%) studies used purely group interventions, and 3 (6%) studies combined both approaches. For practical purposes, henceforth, we will refer only to 2 categories—“group interventions” (consisting of purely group interventions and interventions combining group and dyadic approaches), and “dyadic interventions” (consisting of purely dyadic interventions). A complete list of studies using group interventions [9, 67-104] are presented in Table S1 in Multimedia Appendix 1, while the list of dyadic interventions [10, 105-113] are presented in Table S2 in Multimedia Appendix 1.

In the next subsections, we analyzed the data listed in Tables S1 and S2 in Multimedia Appendix 1 in greater detail to show technology trends in the use of multiuser interventions in mental health care. All the descriptive statistics are calculated from the entirety of 49 studies, adding up to 100%.

### Hardware and Software Used in the Studies

**Figure 2:**
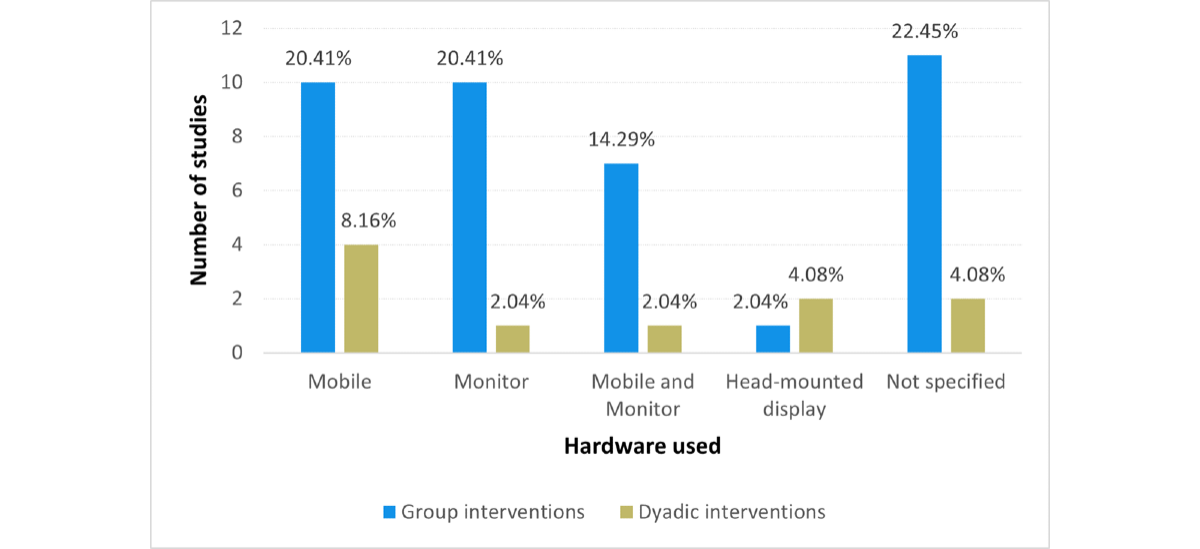
Hardware used in reviewed studies.

In terms of the hardware devices, most of the multiuser interventions used mobile phones (14/49, 29%), computer monitors (11/49, 22%), or their combination (8/49, 16%). Some of the studies did not specify devices used by the target population (13/49, 27%). Importantly, out of 49 studies, only 3 (6%) studies used HMDs (VR headsets). The comparison of the hardware used in the group and dyadic interventions is shown in Figure 2.

**Figure 3:**
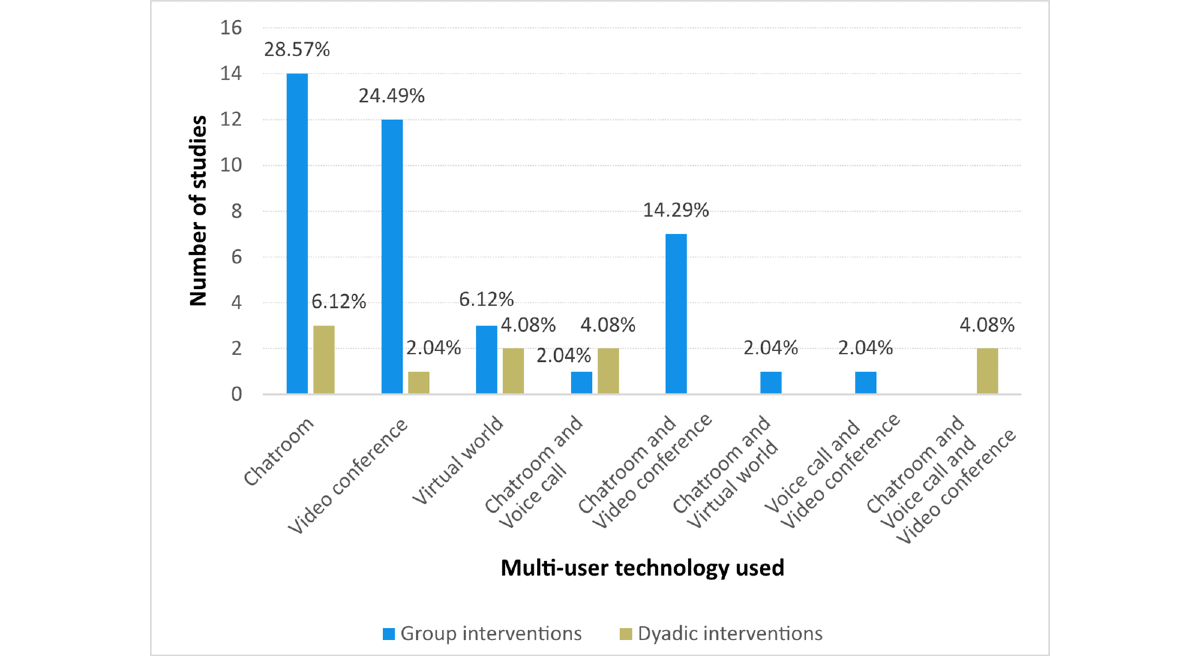
Multiuser technology used in reviewed studies.

In terms of technological tools, the reviewed studies used various multiuser platforms. Some of the studies used only one technology while others used a combination of technologies. For cases where only one technology was used, chat rooms (text form of communication, social network groups, and forums) were used in 17 of 49 (35%) studies, voice calls were not used, videoconferences were used in 13 (27%) studies, and the most advanced technology of virtual worlds was used only in 5 (10%) studies. In the other 14 (29%) studies, the combination of multiple technological tools was used. The comparison of the multiuser technology used in the group and dyadic interventions is shown in Figure 3.

### Type of Support Provided in the Studies

**Figure 4:**
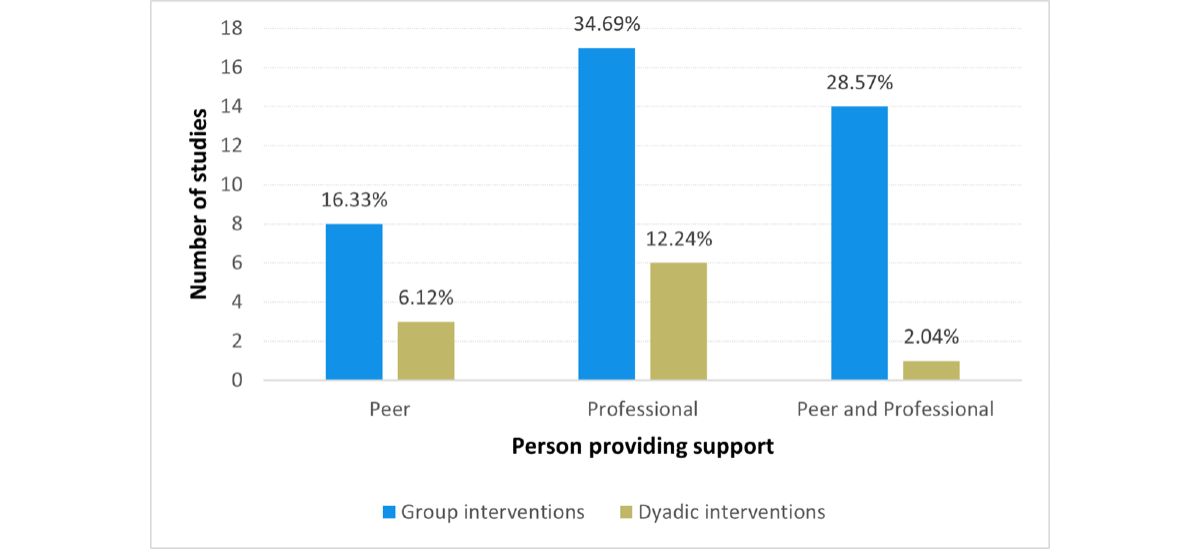
Interpersonal source of support in reviewed studies.

When comparing who moderated the interventions, the studies usually used trained professionals to lead the interventions (23/49, 47%), peer support was used in a few studies (11/49, 22%), while other studies used a combined approach (15/49, 31%). The comparison of the interpersonal source of support in the group and dyadic interventions is shown in Figure 4.

**Figure 5:**
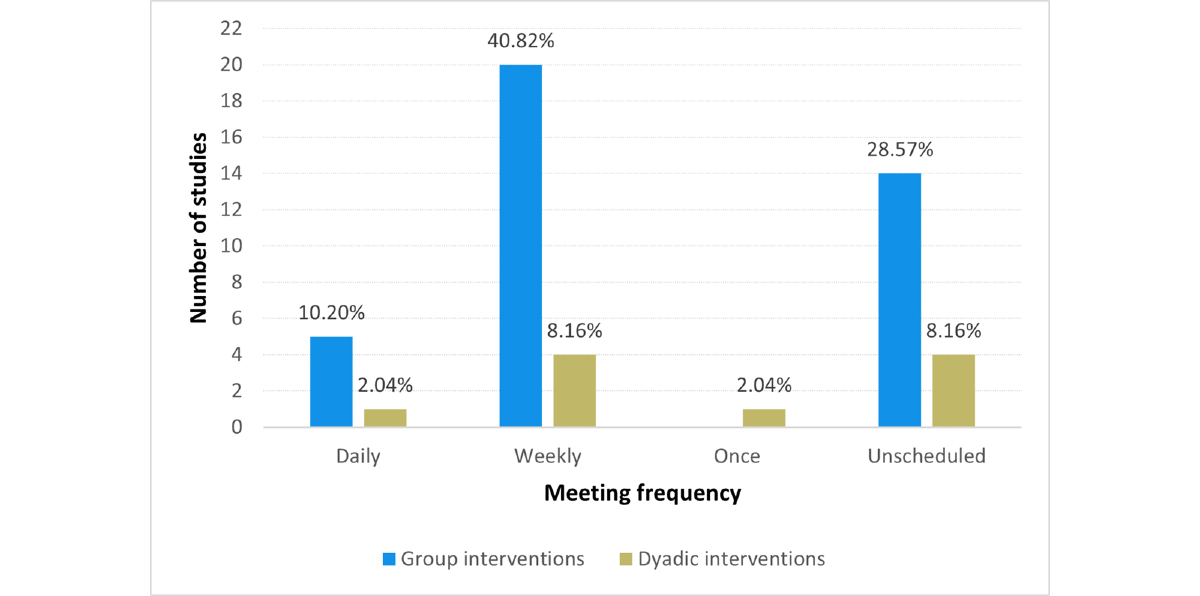
Meeting frequency in reviewed studies.

In terms of frequency, the digital interventions allowed variable (both synchronous and asynchronous) communication, combining multiuser interventions with self-care mobile health apps in some cases. Therefore, we refer only to the frequency of multiuser interventions specifically. The interventions were conducted either daily (6/49, 12%), weekly (24/49, 49%), or in an unscheduled or asynchronous manner allowing the participants to connect at any time—mostly in cases of support chats (18/49, 37%). In addition, there was 1 study that used the group intervention only once (1/49, 2%). The comparison of the meeting frequency in the group and dyadic interventions is shown in Figure 5.

### Clinical Methods and Outcome Measures

Most of the studies were aimed at support groups and stress reduction in different age and target groups. The portion of peer-supported and professional-supported intervention groups is described in detail in the previous paragraph and Figure 4. The interventions that were led by professionals typically involved clinicians, psychotherapists, facilitators, or coaches. The interventions that allowed peer support usually combined this approach with a professional-led component of the intervention.

When led by professionals, the reviewed interventions typically applied CBT approach adapted for online group therapy or other approaches like dialectic behavioral therapy, art therapy, or hypnosis. The peer support groups typically focused on parenting, addiction, and stress reduction methods.

The study designs used in the reviewed records were mostly randomized controlled trials (17/49, 35%), pilot or feasibility studies (10/49, 20%), quasi-experimental studies (8/49, 16%), and qualitative research (9/49, 18%). Out of 49 studies, 2 (4%) studies combined quantitative and qualitative approaches in mixed methods design, and 3 (6%) other studies were in the form of a report, an exploratory study, and an open trial.

The reviewed studies focused on various categories of target populations (similar for group and dyadic interventions) while addressing topics relevant to them. These prevalently included pregnant women or nursing mothers (covering topics such as parenting, self-esteem, or self-efficacy); adolescents, young adults, and university students (covering topics such as stress prevention and reduction); patients with eating disorders, depression, or post-traumatic stress disorder (psychoeducation and symptom reduction); and patients with addiction disorders (eg, smoking cessation). The less represented target groups included minorities (eg, lesbian, gay, bisexual, transgender, intersex, queer or questioning, asexual [LGBTQA+]), patients with psychotic disorders (recovery), and children with autism spectrum disorders (training of social interactions).

The sample size across studies ranged from 3 to hundreds of participants depending on the type of the study (qualitative vs quantitative). The size of different intervention groups also varied, but in most studies, the groups led by a professional included 2 to 10 participants present synchronously. When the intervention was asynchronous, or only peer support was provided, the group size was not always specified or corresponded to the size of the whole sample.

Regarding the outcome measures used, most group and dyadic studies used scales for depression and anxiety, social support, self-esteem and coping, and a variety of stress measures (including physiological markers), and health scales. Some of the studies used additional (or as a main tool in the case of qualitative studies) qualitative interviews with participants. Feasibility studies often included measures of engagement and acceptability of the tested interventions (user experience).

In terms of the clinical effectiveness of the tested digital multiuser interventions, the randomized controlled trials supported the usefulness of these interventions, reporting overall positive results. When compared with waitlists or minimal care, the interventions showed substantial symptom reduction (eg, depression and anxiety) using videoconferencing on mobile phones or computer monitors [74,107,108]; using chat rooms (on computers) [111]; or using a combination of a chat room, voice call, and videoconference either using mobile phones [108] or unspecified hardware [110]. The records showed comparable effects of multiuser interventions in response to standard procedures in a study using videoconference (on computer monitors) [78] and in a study using chat rooms and voice calls (on mobile phones) [105]. Long-term benefits were observed in 3 studies using chat rooms either on mobile phones [76], mobile phones and computers [101], or unspecified hardware [106]. However, some studies reported only partial symptom reduction. This was the case across studies using various technologies, ranging from chat rooms on mobile phones [83], voice calls and videoconferencing on mobile phones [91], and virtual worlds visualized on computer monitors [52,94]. Few studies using chat rooms were unable to confirm effects in the targeted outcomes due to missing or limited data when used on computer monitors [86], mobile phones [97], or using unspecified hardware [95].

## Discussion

### Principal Findings

New technologies allowing multiuser communication and interaction offer innovative solutions for delivering mental health care remotely. They play a crucial role in addressing social isolation and mental health disparities by providing accessible, culturally sensitive, and innovative group-based mental health care to be conducted virtually while overcoming barriers related to geographical distance and mobility.

Overall, the technological innovations leading to a transition from chat rooms (including social network sites) to voice calls, videoconferences, and later to immersive virtual environments, reflect the ongoing evolution of internet-based communication technologies, driven by advancements in internet connectivity, software development, and user demands for richer and more engaging virtual interactions with other humans. This is also reflected in the use of multiuser technologies in clinical research focused on mental health reviewed in this paper.

### Benefits of Digital Multiuser Interventions

The reviewed technologies enable various forms of mental support, ranging from general peer support groups and peer-led interventions to therapist-led group sessions that provide a convenient and accessible alternative to traditional in-person services. Interventions using freely available web-based tools or existing mobile health applications typically require fewer resources than in-person meetings, making them more affordable for organizations and participants.

Online therapy meetings also eliminate geographical barriers, allowing people from different locations to connect easily. Participants can join group sessions from the comfort of their homes, making it more convenient for patients while eliminating physical transportation barriers [84,94,102] (eg, rural environments, older people, and those with a physical disability), overcoming other limitations and stressors created by the mental conditions that should be addressed in the therapy (eg, agoraphobia or social phobia). In some studies, participants provided qualitative feedback and one of the advantages mentioned was that digital communication helps them overcome the fear and stigma of judgment when discussing mental health issues and other sensitive topics. They also perceived the digital platform as facilitating personal reflection [67]. It was also suggested that online group-based interventions and support groups foster community and peer support, allowing participants to connect with others who share similar experiences and challenges. Peer-led support online groups can be especially beneficial, providing a safe space to share experiences, exchange coping strategies, and receive peer validation and encouragement, as needed.

Moreover, online platforms can be designed to meet the cultural and linguistic needs of minority populations, offering support groups and therapy sessions facilitated by professionals who understand their unique experiences and backgrounds. Culturally sensitive interventions [75,89,90] can help reduce stigma and increase engagement in mental health care among marginalized communities.

All these factors explain why many research studies report good acceptability and usability [67,84,85] of multiuser technologies in the target populations, potentially increasing the adherence of patients. By leveraging these technologies, mental health care providers can bridge the gap in access to services and support the well-being of many underserved populations.

### Disadvantages of Digital Multiuser Interventions

There are several disadvantages to digital interventions. These are related to the unwanted effect that technology might have on participants, but there are also unique issues tied to their development and use by health care providers. Some study participants expressed that they would have preferred face-to-face meetings [84,94] and considered virtual settings to be uncomfortable. Indeed, online group interventions are not the same as face-to-face meetings. They present some specific obstacles and challenges that should be compensated for or acknowledged as weaknesses [61].

The most crucial factor undermining the therapeutic efficiency of online interventions could be the absence of body-to-body interaction [61] and limited nonverbal communication. Internet-based meetings often lack the richness of face-to-face interactions, and participants miss out on many nonverbal cues, such as body language and facial expressions (especially in chat rooms and voice calls).

Participants also highlighted various technical issues with the hardware or software. Connectivity problems (network or phone access issues), unclear audio or video, audio and video glitches, and other technical difficulties can easily disrupt the flow of online meetings and hinder effective communication [85].

As the intervention can be carried out from anywhere, it lacks a controlled environment, so background noises often interrupt the meetings [85]. Furthermore, as the meetings happen often at the patients’ homes, they may be more prone to everyday distractions, not focusing fully on the meeting, leading to decreased engagement. Overall, building rapport and fostering a sense of togetherness can be more challenging in virtual spaces compared with in-person interactions.

There are also serious security concerns regarding any treatment happening online, as internet-based meetings may be vulnerable to privacy breaches and security threats, especially when using commercial software without adequate safeguards.

It is also important to add that these technologies, especially the immersive ones, require a new set of skills from health care workers. The therapist must be knowledgeable about the software, potential issues, and how to solve them, as having IT support might not be possible during private sessions.

Internet-based chats, voice calls, and videoconferencing have become so common that it could be expected that each health care worker and patient can use or can learn to use them. However, setting up immersive devices and accessing virtual worlds can be much more challenging for new users not familiar with this technology. This can lead to the patients focusing more on the technology than the session itself.

Finally, there are considerable problems to overcome with regard to developing or hosting one’s own multiuser platform. Using existing solutions, such as social platforms, dedicated software, games, or videoconferencing can be costly, but it also raises questions about privacy, data collection, and data retention, which need to be discussed. These existing technologies could be more suited for peer support groups with no affiliation to the health care providers.

For these reasons, many of the reviewed studies have built new applications and solutions, but such development often requires initial and ongoing investments in terms of software development and the cost of maintaining servers. Any application, whether on a mobile phone or in VR, which allows multiuser engagement, requires a backend service to transfer data between users constantly. The applications must also be regularly updated to abide by new security standards or with new browsers and operating systems being released. All this can be a complicated process that is not easily implemented within the organizational structure of health care institutions.

### Increasing Immersion in Multiuser Interventions

It seems that multiuser digital interactions are slowly making their way into mental health care. This is apparent from our findings, which demonstrates that over the years the number of studies using these technologies is increasing. We have observed that chat rooms and videoconferencing still represent the most prevalent methods used in mental health care performed in groups, at least represented in research studies. The popularity of chat rooms is probably due to their low barrier of entry and because they allow both synchronous and asynchronous communication. This makes them especially useful in design protocols where self-care methods and psychoeducation tools are combined with on-demand support from peers or professionals [99].

However, purely text-based online support does not offer many important aspects of in-person group interventions, such as speech tonality or facial expressions. These can be achieved using videoconferencing software, which has become very popular in health care. Participants can hear and see each other and react to a multitude of nonverbal cues. It allows participants to freely express themselves, which may increase interactions and invite active participation. As videoconferencing is typically synchronous, it also promotes a sense of presence in the group. For these reasons, along with relative ease of use, videoconferencing seems to be highly prevalent in group therapeutic settings.

By contrast, videoconferencing is still just a series of individual camera streams and lacks the spatial and interactive aspects of human communication (eg, we cannot look directly at the person we are talking to in the group).

In contrast, multiuser virtual world environments enable us to create a common space that the participants share in the same moment, allowing for greater engagement or more natural conversations. Virtual worlds offer more naturalistic social interactions. People can move toward or away from one another, and they can look at each other while talking. The software can incorporate hand gestures or full-body tracking. However, immersive visualization of the avatar’s body might present an obstacle if not correctly synchronized with the participant’s body movements. This can be a problem for full-body avatars as feet are generally not tracked by commercial HMDs. It has been shown that half-body avatars with head and hand tracking present a good option for social interactions and are able to induce good (co)presence in the multiuser VR environment [109,114].

Some current VR headsets, such as Meta Quest Pro or Vision Pro, also allow recognition of facial expressions and eye movements (gaze direction and blinking), which are then translated to the avatar.

These immersive features have been shown to enhance a sense of social presence [100] or copresence [109]. Participants reported feeling like being in the same room with others, even though they met only in a virtual environment [84,100]. This may potentially increase the feeling of togetherness and thus provide an experience similar to the one provided in face-to-face group communication.

In addition, VR worlds (eg, Second Life [115] or Innerworld [116]) allow participants to customize their avatars, fostering their creativity and self-expression that could occur during in-person interactions [102], while guaranteeing anonymity, if needed. This customization allows participants to stay fully anonymous by selecting a generic avatar and even changing their real voice to an artificial one. On the contrary, they can convey selected personal aspects about themselves, such as sex, race, or age through the avatar’s physical features and clothing. Therefore, the virtual worlds protect the participants’ identity and privacy while keeping some of the nonverbal-communication cues still available [109].

Importantly, the anonymity given by the avatar allows participants to disclose emotions and concerns more freely and participate in social interactions with minimal risk of rejection [100]. However, we need to be mindful of the Proteus effect, which shows that the behavior in virtual worlds tends to mimic the characteristics of the virtual avatar chosen [117]. Herrera et al [114] reported that the behavioral realism of avatars used in the virtual environment also affects their nonverbal behavior in the physical world. This factor, often omitted in the current studies, could have both beneficial effects (eg, in increasing self-confidence and courage) and negative influence (eg, an increase in antisocial behavior toward other members of the group), and should be considered in the future usage of VR avatars in multiuser clinical interventions.

Finally, the professional or peer moderating the group session also has perfect control over the content of meetings in virtual worlds, often missing in other internet-based communication tools [61], enabling them to create a safe and supportive environment for each participant.

However, some constraints should be considered in VR interventions, mainly due to technical demands, as virtual worlds may require specialized hardware and software to access them. These technical requirements could be a barrier for some individuals and are probably why, despite many advantages, VR interventions are not so frequently used in clinical settings yet. Entering the virtual world does not require a VR headset (HMD) in general, as it can be presented on a traditional computer screen, at the expense of a lower immersion level. However, accessing virtual worlds using any device can be challenging for new users not familiar with this technology (eg, almost a third of the new users in the study by Nosek et al [102], described Second Life as “somewhat difficult” to learn).

In contrast, highly immersive virtual devices such as HMDs, provide a higher sense of presence for the participants that could facilitate the therapeutic process. An observational study using the Innerworld VR application [116] suggested that a higher level of immersion with HMD could potentiate the anticipated symptom changes in contrast to devices with lower immersion. However, higher immersion might be potentially associated with some adverse effects, such as cybersickness symptoms (eg, headache or dizziness). A recent systematic review analyzed the side effects reported using the Simulator Sickness Questionnaire in 55 documented research articles on VR clinical interventions and concluded that side effects (related mainly to disorientation, nausea, and oculomotor disturbances) were reported more frequently with HMDs than in desktop systems [118]. Another systematic review conducted on 73 studies with VR and AR interventions shows that 45 of these studies failed to mention or measure adverse effects completely [119], which is alarming. Cybersickness should be evaluated using standardized measures and minimized with regard to the potential negative impact (eg, worsening clinical symptoms or an increased fall risk [119]). The benefits of the applied intervention should always outweigh the risks and challenges associated with the technology. Immersive group interventions in anxiety disorders should also be monitored for potential ambiguities in cybersickness evaluation as these symptoms might be mediated by provoked anxiety [120], and standardized measures, such as Simulator Sickness Questionnaire should be adjusted in this target group accordingly, as suggested by Bouchard et al [121].

By contrast, advanced HMD devices could enable stimulation through multiple sensory modalities, by incorporating features, such as haptic feedback, spatial audio, and realistic environmental elements that can further enhance immersion and user engagement which should be addressed in future studies. Moreover, the shift toward MR technology enables us to benefit from social collaboration and interactions within the comfort of familiar surroundings. This combination of virtual and physical environments prepares the ground for highly social and colocated experiences, which could be applied not only remotely but also to in-person therapies [122].

Overall, immersion in the context of multiusers VR interventions could enhance social interactions and collaboration among participants [9]. By creating shared virtual spaces where users can interact and engage with each other, multiuser VR applications can foster a sense of community and support among individuals seeking mental health interventions. Interventions in virtual worlds are also reported to be more enjoyable [94].

However, the accessibility of this fast-developing immersive technology to clinical facilities and clinical populations represents a huge disadvantage. Despite the notable reduced financial costs of acquiring HMDs, the rapid obsolescence of the technology from the perspective of manufacturers constantly creating new devices greatly complicates the development of clinical applications. Developers must place considerable emphasis on multi-platform software solutions compatible with different brands and iterations of headsets, which is even more challenging in multiuser solutions.

### Limitations

The main study limitation is the inability to synthesize the selected records. The variability of target groups, study designs, methods, and outcome measures used in the reviewed records prevented us from a rigorous synthesis of the reported studies, which were not easily comparable. As our main objective was to address the rate of occurrence or popularity of multiuser digital interventions for mental health care, we did not focus in detail on the efficiency of individual applications and the synthesis of their clinical outcomes. The large variability could also lead to a publication bias. When so many different technologies and research methodologies are employed, some studies might observe positive results by chance, and without replication, such findings might not be refuted. We have observed most of the studies reporting positive outcomes, but we cannot be certain if this is due to the efficiency of the method, or due to a publication bias skewed toward positive results.

Another limitation is related to the query used for the database search of the selected records. The inconsistency in the terminology used in the reviewed articles, particularly in the context of “multiuser” technologies, creates a potential negative selection bias that could lead to certain publications not being registered in our search. For example, the term “cognitive behavioral immersion” has been used in a few studies testing the Innerworld application [21,116] not included in the systematic search.

### Conclusions and Future Directions

Overall, participants in the multiuser digital interventions experienced positive emotional, social, and clinical outcomes. Although internet-based group meetings and virtual worlds offer unique advantages and opportunities for social connections, they also present challenges that must be addressed to ensure effective communication and engagement during group interventions. Balancing the benefits of accessibility and convenience with the limitations of technology and social interaction remains a key consideration in leveraging these platforms for mental health support and peer mediation.

To allow for systematic research, future studies should follow certain principles. One of the most important factors is a clear unification of terminology for multiuser interventions. Detailed reporting of methods is also important, especially with regard to the applied devices and procedures used during these interventions, including the frequency and synchronicity of sessions and standardized outcome measures, which then allows for valid evaluation of observed effects in case of feasibility studies and clinical trials.

As technology evolves, we can expect further innovations in how people connect and collaborate in virtual spaces, shaping the future of remote communication and collaboration. On the basis of the technological progress in VR and MR devices and multiuser virtual environments, the potential benefits of these platforms could be used in mental health care in the near future. Recent developments have shown promise in leveraging the immersive nature of VR to create engaging and effective mental health interventions in multiuser setups. In the future, virtual spaces tailored to specific mental health conditions are likely to be developed. In group VR therapy, an environment where all patients or clients engage in a shared task could be used, similar to individual VR-based exposure therapy, wherein patients with social phobia might practice public speaking in a virtual auditorium with other patients being able to observe and interact, thus learn from each other’s experiences and strategies and experience a sense of support. Current applications also do not offer many opportunities and benefits of virtual spaces, such as using stimuli presented in the environment (eg, a virtual whiteboard). However, the technology must be studied in detail with regard to its limitations and challenges mentioned previously, as these may lead to a reduction or complete suppression of the expected therapeutic effects.

Future research studies addressing the feasibility and effectiveness of VR-based multiuser interventions should also address some of the gaps identified in this review. Particularly, neither the effects of customized avatars compared with real identities nor the Proteus effect was studied in reported clinical studies. Moreover, various levels of immersion used to visualize virtual environments were not directly compared in previous studies. Thus, it is not clear whether immersive VR provides clear benefits with regards to the clinical effectiveness of multiuser interventions that would diminish or outweigh any barriers that this technology certainly has so far.

The introduction of AI chatbots represents the next logical step in digital interventions. AI technology is not yet sufficiently advanced to be fully comparable to human communication, especially when simulating experts and peers. Errors and glitches in verbal and nonverbal communication, and other limitations, such as missing personalization or simulated emotions and empathy [123], might affect intervention outcomes, especially in terms of adherence rate and long-term benefits [124]. Nevertheless, further technological developments in this area will surely lead to significant advances over time, especially in dyadic communication agents providing scalable and accessible first-contact intervention in mental health care [125]. In addition, there will surely be an emphasis on the creation of ethical frameworks to guide the development and use of these technologies and to address issues, such as patients’ privacy or potential biases in AI algorithms [126].

## Appendix

Multimedia Appendix 1 Complete list of 49 reviewed studies using group digital interventions.

Multimedia Appendix 2 PRISMA checklist.

## Abbreviations

AI: artificial intelligence
AR: augmented reality
CBT: cognitive behavioral therapy
HMD: head-mounted display
LGBTQA+: lesbian, gay, bisexual, transgender, intersex, queer or questioning, asexual
MR: mixed reality
PRISMA: Preferred Reporting Items for Systematic Reviews and Meta-Analyses
VR: virtual reality

## Data Availability

The datasets generated during and analyzed during this study and code used to generate them are available in the GitHub repository [127].
