# Supplementary material for: The Journey From Nonimmersive to Immersive Multiuser Applications in Mental Health Care: Systematic Review
Source: J Med Internet Res. 2024 Nov 7;26:e60441. doi: 10.2196/60441 (PMC11582485; doi:10.2196/60441)
Supplement: Multimedia Appendix 1 [file jmir_v26i1e60441_app1.pdf]

Table S1. Studies using group digital interventions for 3 or more users.

Study, year	Title	Multiuser technology	Hardware	Peer or expert	Meeting frequency	Target group	Sample size, N	Demography	Study type	Control group	Intervention—short description	Outcome measures
Ronen et al [67], 2024	Acceptability and Utility of a Digital Group Intervention to Prevent Perinatal Depression in Youths via Interactive Maternal Group for Information and Emotional Support (IMAGINE): Pilot Cohort Study	Videoconference, chat room	Not specified	Expert	Unscheduled	Pregnant or <180 days postpartum women, aged 16-24 years during pregnancy	10	10 female participants, median 17.9 (IQR 17.4-21.7)	Pilot study	No	IMAGINE—Interactive Maternal Group for Information and Emotional Support	Abbreviated 12-item version of the Social Support Behavior (SSB); Perceived Stress Score (PSS-4); In-Depth Interviews (IDIs)
Pokowitz et al [68], 2024	Mood Lifters for Graduate Students and Young Adults: A Mixed-Methods Investigation into Mechanisms of Change in Online Group Therapy	Videoconference	Mobile, monitor	Peer	Weekly	The graduate student and young adult populations	138 (79 students and 59 adults)	130 female participants, 6 male participants, 2 others; students mean age 25.7 (SD 2.68 y), range 22-32; adults: mean age 27.3 (SD 3.19 y), range 22-33	Mixed methods study	Yes	Mood-lifters—peer-led group therapy program in which individuals are introduced to content and skills across biopsychosocial domains, including sleep, body, mind, mood, behavior, and social content	Experience with Mood Lifters; Therapeutic factor inventory-8; Group Climate Questionnaire—Engagement Subscale; Attendance
Penwell et al [69], 2024	Traditional versus virtual partial hospital program for eating disorders: Feasibility and preliminary comparison of effects	Videoconference	Not specified	Expert	Daily	Patients with eating disorders	140 (70 virtual interventions and 70 traditional interventions)	Virtual: mean age 23.1 (SD 10.4 y), cis-woman, 98.6%; trans-man, 1.4%; traditional: mean age 23.3 (SD 9.3 y), cis-woman, 95.7%; nonbinary 4.3%	Feasibility study	Yes	Virtual Partial Hospital Programme (PHP) for eating disorders	Eating Disorder Examination-Questionnaire (EDE); The Overall Anxiety Severity and Impairment Scale (OASIS); The Anxiety Sensitivity Index (ASI); The Brief Experiential Avoidance Questionnaire (BEAQ); The Southampton Mindfulness Questionnaire (SMQ); The Progress Monitoring Tool for ED (PMED); BMI; discharge status; disorder diagnosis
Conroy et al [70], 2024	Learning from Adolescents and Caregivers to Enhance Acceptability and Engagement Within Virtual Dialectical Behavior Therapy for Adolescents Skills Groups: A Qualitative Study	Videoconference	Not specified	Expert	Weekly	Adolescents (with mental health problems) and caregivers	33	Mean age 15 (SD 1.39 y), age range 13-18 y; 22 female, 8 male, 1 nonbinary participant	Qualitative study	No	Telehealth Dialectical Behavior Therapy for Adolescents (DBT-A) program	Focus groups
Zimmerman et al [71], 2023	Telehealth treatment of patients with major depressive disorder during the COVID-19 pandemic: Comparative safety, patient satisfaction, and effectiveness to pre-pandemic in-person treatment	Videoconference	Not specified	Expert	Daily	Major Depressive Disorder (MDD)	836 (294 virtual and 542 control)	Virtual: mean age 38.27 (SD 14.17 y); control: mean age 39.07 (SD 15.26 y) | 592 female participants, 217 male participants, 25 other	Report	Yes	Telehealth treatment for MDD	Depression subscale of a modified version of the Remission from Depression Questionnaire (RDQ-M); Self-administered measures of patient satisfaction, symptoms, coping ability, functioning, and general well-being; Clinically Useful Patient Satisfaction Scale (CUPSS)

Xie et al [72], 2023	Building Emotional Awareness and Mental Health (BEAM): an open-pilot and feasibility study of a digital mental health and parenting intervention for mothers of infants	Videoconference, chat room	Mobile, Monitor	Peer, expert	Weekly	Mothers with elevated depression scores with children 6-17 months old	41	Mean age 30.83 (SD 4.86 y), age range 19-39 y | 46 female participants	Feasibility study	No	Digital mental health and parenting intervention for mothers of infants	The mHealth App Usability Questionnaire (MAUQ); Patient Health Questionnaire (PHQ-9) for depression symptoms; Parenting Stress Index—Short Form (PSI/SF); Generalized Anxiety Disorder 7-Item Scale (GAD-7); Child Behaviour Checklist (CBCL)
Wright et al [73], 2023	A virtual mother-infant postpartum psychotherapy group for mothers with a history of adverse childhood experiences: open-label feasibility study	Videoconference	Monitor	Peer, expert	Weekly	Mothers with a history of adverse childhood experiences (ACEs) during the postpartum period.	31	Mean age 36.5 (SD 3.9 y) | >95% female participants	Feasibility study	No	MOMBABY—live video-based 12-wk interactive psychotherapy group focused on maternal symptoms and maternal-infant relationships	Edinburgh Postnatal Depression Scale (EPDS); Generalized Anxiety Disorder-7 scale (GAD-7); PTSD Checklist for DSM5 scale (PCL-5); Difficulties in Emotion Regulation Scale (DERS); Parenting Stress Index – Short Form (PSI-4-SF); Parental Reflective Functioning Questionnaire (PRFQ)
Wang et al [74], 2023	Randomized controlled pilot study of feasibility and effectiveness of peer led remote Mindfulness-Based Art Workshops on stress, anxiety, and depression in medical students	Videoconference	Monitor	Peer	Weekly	Medical students—mindfulness program for stress, anxiety, and depression	24 (12 interventions and 12 control)	Intervention: mean age 24.8 (SD 1.8 y); control: mean age 23.3 (SD 1.2 y) | 22 female participants	Randomized controlled trial	Yes	Peer-led remote Mindfulness-Based Art Workshop on stress, anxiety, and depression	State Trait Anxiety Inventory (STAI); National Institutes of Health (NIH) Toolbox Perceived Stress survey
Hunt et al [75], 2023	South African university students' experiences of online group cognitive behavioural therapy: Implications for delivering digital mental health interventions to young people	Videoconference, chat room	Monitor	Peer, expert	Weekly	University students	125	Mean age 21.96 (SD 4.5 y) | 85% female participants	Qualitative study	No	Online Group CBT intervention	Semistructured interview; Analysis of experiences of the online GCBT intervention to understand Acceptability of the "hybrid" format; Barriers and facilitators to engagement; Reflections on the digital intervention; Contextual and cultural appropriateness of the intervention
Hollis et al [76], 2023	Online remote behavioural intervention for tics in 9-to 17-year-olds: the ORBIT RCT with embedded process and economic evaluation	Chat room	Monitor	Expert	Unscheduled	Children aged 9-17 years with Tourette syndrome or chronic tic disorder	224 (112 interventions and 112 control)	Mean age 12 y | 177 male participants	Randomized controlled trial	Yes	Online therapist- and parent-supported therapy for young people with tic disorders, delivered via Barninternetprojektet—a Swedish web-based digital platform	The primary clinical outcome was tic severity, secondary were tic-related impairment, behavioral and emotional difficulties, global improvement, QoL, service use, and treatment credibility and satisfaction were also obtained
Gerson et al [77], 2023	Patients' experiences with virtual group gut-directed hypnotherapy: A qualitative study	Videoconference	Not specified	Expert	Weekly	People with gastrointestinal conditions	21	Mean age 47.7 (SD 16.3 y), age range 29-77 y |16 female participants, 4 male participants, 1 other	Qualitative study	No	Virtual, group-based, gut-directed hypnotherapy (GDH)	Qualitative interviews conducted one-on-one with patient
Finnerty et al [78], 2023	Online group music therapy: proactive management of undergraduate students' stress and anxiety	Videoconference	Monitor	Expert	Weekly	Healthy university students—prevention	84 (28 receptive music therapy, 18 active music therapy, 18 verbal therapy, 20 control)	Mean age 20 y, age range 18-24 y | 15 male participants	Randomized controlled trial	Yes	Online group music therapy (including songwriting, singing, lyric analysis, and verbal processing)	State and Trait Anxiety Inventory (STAI-S); Likert stress scale; Ten Item Personality Inventory (TIPI); GOLD-MSI; World Health Organization Quality of Life (WHO-QOL); stress measures collected in week 1 and 6 (PSS and cortisol) and HRV

Brownstone et al [79], 2023	Sharing Lived Experience: Describing a Virtual Counselor-Facilitated LGBTQ plus Support Group for Disordered Eating	Video conference	Not specified	Peer, Expert	Weekly	Sexual and gender minority (SGM) individuals with disordered eating	27	Not applicable	Quasi-experimental study	No	LGBTQ+ Virtual Support Group for individuals with eating disorders	Survey that included open-ended questions
Zaccari et al [80], 2022	Synchronous Telehealth Yoga and Cognitive Processing Group Therapies for Women Veterans with Posttraumatic Stress Disorder: A Multisite Randomized Controlled Trial Adapted for COVID-19	Video conference, Cha troom	Mobile, Monitor	Expert	Weekly	Women veterans with post-traumatic stress disorder (PTSD) secondary to military sexual trauma	132 (28 virtual and 140 control)	Pacific Northwest: mean age 47.5 (SD 11.7 y); Southeast: mean age 48.43 (SD 11.2 y) | 117 female participants	Randomized controlled trial	Yes	Synchronous telehealth yoga and cognitive processing group therapy for women veterans with PTSD	PTSD Scale for Diagnostic and Statistical Manual of Mental Disorders Fifth Edition (CAPS-5); Self-report measures of health, and psychophysiological markers of stress; Qualitative data collected through interviews; Heart Rate Variability (HRV); ECG; Blood samples
Mi et al [81], 2022	Intraindividual, Dyadic, and Network Communication in a Digital Health Intervention: Distinguishing Message Exposure from Message Production	Chatroom	Mobile	Peer	Unscheduled	Addiction recovery	268	Mean age 42.33 (SD 0.77 y) | 147 female participants	Quasi-experimental study	No	Seva ("selfless caring") - mobile health app for addiction recovery	In-system communication actions were categorized to reflect intra-individual, dyadic, or network communication; pages were categorized according to whether they represented message consumption (content exposure) or message composition or editing (content production); measures of participant's total number of interactions (or "clicks")
Mendelson et al [82], 2022	Remote group therapies for cognitive health in schizophrenia-spectrum disorders: Feasible, acceptable, engaging	Video conference	Mobile, Monitor	Expert	Weekly	Schizophrenia-spectrum disorders	28	Mean age 33.9 (SD 11.8 y) | 9 female participants, 12 male participants	Feasibility study	No	Remote group therapies for cognitive health in schizophrenia-spectrum disorders using Zoom for Healthcare	Feasibility, acceptability and engagement assessment
Kruzan et al [83], 2022	Use of a Mobile Peer Support App Among Young People With Nonsuicidal Self-injury: Small-scale Randomized Controlled Trial	Chatroom	Mobile	Peer	Unscheduled	Young people with Nonsuicidal Self-Injury (NSSI)	131 (67 interventions and 64 control)	Mean age 20.32 (SD 2.52 y) | 89 female participants, 24 male participants, 15 nonbinary, 3 other	Randomized controlled trial	Yes	Talklife - Mobile Peer Support App for Young People With Nonsuicidal Self-injury	Self-injury form checklist from NSSI-AT; two items adapted from Alexian Brothers Urge to Self-Injure Scale; Readiness Ruler for readiness to change; disclosure items from NSSI-AT; NSSI-AT Treatment Experiences items; Attitudes Toward Seeking Professional Psychological Help Scale
Fell et al [84], 2022	Acceptability of A Virtual Mind-Body Group Intervention for Teen Siblings of Children with Autism Spectrum Disorder	Video conference	Not specified	Expert	Weekly	Teenage (age 14-17 years) siblings of children with autism spectrum disorder	35	Mean age 15 (SD 1.2 y) | 20 female participants	Mixed methods study	No	Sib-Chat - Virtual Mind-Body Group Intervention for Teen Siblings of Children with Autism Spectrum Disorder	Quantitative and qualitative post-intervention feedback survey with closed-ended (on 3-4-point scale) questions and open-ended questions about the intervention in general to assess acceptability and efficacy
El Ayadi et al [85], 2022	A Mobile Education and Social Support Group Intervention for Improving Postpartum Health in Northern India: Development and Usability Study	Voice call, Chat room	Mobile	Peer, expert	Weekly	New mothers	29	29 female participants, median 25 (IQR 24-28)	Feasibility study	No	A Mobile Education and Social Support Group Intervention for Improving Postpartum Health	Intervention feasibility and acceptability

Ali et al [86], 2022	Recruitment, adherence and attrition challenges in internet-based indicated prevention programs for eating disorders: lessons learned from a randomised controlled trial of ProYouth OZ	Chat room	Monitor	Peer	Weekly	Young adults at risk for eating disorders	50 (34 interventions and 16 control)	Mean age 21.3 (SD 2.4 y) | 47 female participants	Randomized controlled trial	Yes	ProYouth OZ - Internet–based prevention program for eating disorders	Eating disorder symptoms
Yeshua-Katz et al [87], 2021	The Role of Communication Affordances in Post-Traumatic Stress Disorder Facebook and WhatsApp Support Groups	Chat room	Mobile, Monitor	Peer	Unscheduled	Military veterans with PTSD and their partners	34	Mean age 47 y, 29-77 y | 13 female participants, 21 male participants	Qualitative study	No	Online support groups (OSGs) for military veterans with PTSD	Qualitative in-depth interviews and survey methods
Dabit et al [88], 2021	Improving social functioning in people with schizophrenia-spectrum disorders via mobile experimental interventions: Results from the CLIMB pilot trial	Videoconference, chat room	Mobile	Peer	Weekly	Patients with schizophrenia Spectrum Disorders (SSD)	24 (intervention =12; control=12)	Mean age 36.6 (SD 10.8 y) | 16 female participants, 11 male participants, 4 nonbinary	Pilot study	Yes	Mobile experimental interventions for social functioning improvement in people with schizophrenia-spectrum disorders	Social Functioning Scale (SFS); abbreviated Quality of Life Scale (aQLS); Positive and Negative Syndrome Scale (PANSS)
Craig et al [89], 2021	AFFIRM Online: Utilising an Affirmative Cognitive-Behavioural Digital Intervention to Improve Mental Health, Access, and Engagement among LGBTQA plus Youth and Young Adults	Video conference, Chatroom	Monitor	Peer, expert	Weekly	LGBTQA+ youth and young adults	96 (intervention=46; control=50)	Mean age 21.17 (SD 4.52 y) | 34 nonbinary, 61 other	Quasi-experimental study	Yes	AFFIRM - CBT 8-session group intervention designed for LGBTQA+ youth	Brief COPE Inventory; 21-item Beck's Depression Inventory-II (BDI-II); Stress Appraisal Measure for Adolescents (SAMA); 12-item Hope Scale (HS); AFFIRM Acceptability Survey; 17-item questionnaire with 4-point Likert responses specific to this research and not standardized
Craig et al [90], 2021	Adapting Clinical Skills to Telehealth: Applications of Affirmative Cognitive-Behavioral Therapy with LGBTQ+ Youth	Video conference, Chatroom	Not specified	Expert	Weekly	LGBTQ+ youth	1 (case study)	1 nonbinary	Qualitative study	No	AFFIRM - CBT 8-session group intervention designed for LGBTQA+ youth	Case study
Abedishargh et al [91], 2021	Effectiveness of Internet-Based Cognitive Behavioral Therapy in Weight Loss, Stress, Anxiety, and Depression via Virtual Group Therapy	Video conference, Voice call	Mobile	Peer, expert	Daily	Obesity- depression, anxiety, and stress in obesity	90 (intervention=30; control=60)	Range 18-30 y (N=29); 31-40 y (N=22); 41-50 y (N=25)	Randomized controlled trial	Yes	ICBT (internet based CBT) virtual group for stress, anxiety, and depression in overweight women	Depression Anxiety Stress Scale (DASS) and BMI
Tsai et al [92], 2020	Effects of a smartphone-based videoconferencing program for older nursing home residents on depression, loneliness, and quality of life: a quasi-experimental study	Video conference	Mobile	Peer, expert	Unscheduled	Nursing home residents	62 (intervention=32; control=30)	CG: mean age 68.95 (SD 11.65 y); IG: mean age 81.07 (SD 8.46 y) | 41 female participants, 21 male participants	Quasi-experimental study	Yes	Smartphone-based videoconferencing program for older nursing home residents on depression, loneliness, and quality of life using the LINE platform	Feelings of loneliness, depressive symptoms and quality of life
Strand et al [93], 2020	Combining online and offline peer support groups in community mental health care settings: a qualitative study of service users' experiences	Chat room	Monitor	Peer	Unscheduled	Various psychiatric diagnoses	14	Focus groups: mean age 45 y, 22-63 y; Interviews: mean age 47 y, 24-67 y | 21 female participants, 0 male participants	Qualitative study	No	ReConnect—internet-based peer support portal for people with long-term mental health problems	Open-ended questions on recovery processes (focus groups)

Robinson-Whelen et al [94], 2020	Promoting psychological health in women with SCI: development of an online self-esteem intervention	Virtual world	Monitor	Peer, expert	Weekly	Spinal cord injury	21 (intervention=10; control=11)	CG: mean 44.00 (SD 13.25 y); IG: mean 50.90 (SD 9.95 y) | 23 female participants	Randomized controlled trial	Yes	Self-esteem intervention in the form of real time group sessions in Second Life—a free online virtual world	Interpersonal Support, Spiritual Growth/Self-actualization, and Stress Management subscales from the Health Promoting Lifestyle profile-II; Emotional/Informational Support subscale of the Medical Outcomes Study Social Support Survey; Generalized Self-Efficacy Scale; Rosenberg Self-Esteem Scale; Center for Epidemiological Studies Depression scale-10 (CESD-10); Patient Health Questionnaire (PHQ-9)
Vogel et al [95], 2019	Smoking Cessation intervention trial outcomes for sexual and gender-minority young adults	Chat room	Not specified	Peer, expert	Daily	Smokers (addiction)	500 (intervention=251; control=249)	54.6% female participants, 44.8% male participants, 0.6% transgender	Randomized controlled trial	Yes	Smoking cessation intervention using Facebook groups	Participants reported smoking status and health risk behaviors at baseline, 3, 6, and 12 mo; usability of the intervention (ie, perceptions of the intervention and treatment engagement) assessed in the intervention group at 3 mo
Tait et al [96], 2019	A Digital Intervention Addressing Alcohol Use Problems (the Daybreak Program): Quasi-Experimental Randomized Controlled Trial	Chat room	Mobile, Monitor	Peer, expert	Unscheduled	Individuals with alcohol-related problems	793 (intervention=398; control=395)	Intervention: mean 40.9 (SD 10.0 y); control: mean 41.0 (SD 10.1 y) | 561 female participants	Quasi-experimental study	Yes	The Daybreak Program: A Digital Intervention Addressing Alcohol Use Problems	Change in alcohol risk; number of standard drinks per week; alcohol-related days out of role; psychological distress; quality of life
Sawyer et al [97], 2019	The Effectiveness of an App-Based Nurse-Moderated Program for New Mothers With Depression and Parenting Problems (eMums Plus): Pragmatic Randomized Controlled Trial	Chat room	Mobile	Expert	Unscheduled	New mothers with depression and parenting problems	133 (intervention=72; control=61)	Intervention: mean 31.1 (SD 5 y); control: mean age 32.2 (SD 4 y) | 133 female participants	Randomized controlled trial	Yes	eMums Plus—App-Based Nurse-Moderated Program for New Mothers With Depression and Parenting Problems	Level of maternal depressive symptoms; quality of maternal caregiving; Parenting Sense of Competence Scale; Nursing Child Assessment Satellite Training Scale
Campbell et al [98], 2019	A Customized Social Network Platform (Kids Helpline Circles) for Delivering Group Counseling to Young People Experiencing Family Discord That Impacts Their Well-Being: Exploratory Study	Chat room	Mobile	Expert	Unscheduled	Young people experiencing family discord with mild to moderate depression or anxiety	105	Mean age 16.2 (SD 2.9 y) | 86 female participants, 10 male participants, 9 other	Exploratory study	No	Kids Helpline Circles - A Customized Social Network Platform for Delivering Group Counseling to Young People Experiencing Family Discord	Social support; anxiety and depression level; self-esteem; qualitative questions on user experience, perceived benefits etc.
Granado-Font et al [99], 2018	Coping Strategies and Social Support in a Mobile Phone Chat App Designed to Support Smoking Cessation: Qualitative Analysis	Chatroom	Mobile	Peer, expert	Unscheduled	Smokers during first 3 months of the smoking cessation	102	Mean age 45.3 (SD 8.9 y) | 43 female participants, 59 male participants	Qualitative study	No	Tobbstop app-Mobile Phone Chat App Designed to Support Smoking Cessation	Content analysis of text messages posted to the chat aimed at the emotions, motivations, and perceived benefits in daily experiences within the process of change
Knowles et al [100], 2017	A pilot study of virtual support for grief: Feasibility, acceptability, and preliminary outcomes	Chatroom, Virtual world	Head-mounted display	Expert	Weekly	Widow(er)s	30 (intervention=18; active control=12)	Mean age 67.0 (SD 1.0 y) | 21 female participants, 9 male participants	Feasibility study	Yes	Virtual support for grief in Second Life	Geriatric Depression Scale; Yearning in Situations of Loss; Sleep Quality Index; Loneliness; Perceived Stress

Galliers et al [9], 2017	Experiencing EVA Park, a Multi-User Virtual World for People with Aphasia	Virtual world	Monitor	Expert	Daily	People with aphasia	20	Mean age 57.8 y | 9 female participants, 11 male participants	Quasi experimental study	No	EVA Park, a Multi-User Virtual World for People with Aphasia	Interactions in the virtual world (coding exercise); video recordings (participant interacting with EVA Park, and simultaneously what was happening in the virtual world); interviews; time log - how long participants spent in EVA Park outside of scheduled sessions
Carolan et al [101], 2017	Increasing engagement with an occupational digital stress management program through the use of an online facilitated discussion group: Results of a pilot randomised controlled trial	Chatroom	Mobile, Monitor	Peer, Expert	Unscheduled	Working people	84	Mean age 41.0 (SD 10.2 y) | 70 female participants	Randomized controlled trial	No	WorkGuru - CBT web-based stress management intervention	Engagement; wellbeing at work IWP; dass-21
Nosek et al [102], 2016	An Internet-Based Virtual Reality Intervention for Enhancing Self-Esteem in Women With Disabilities: Results of a Feasibility Study	Virtual world	Not specified	Expert	Weekly	Women with disabilities	19	Age range 24-61 y | 19 female participants, 0 male participants	Feasibility study	No	Self-esteem 7-session interactive group intervention in SecondLife, using avatars with voice and text communication	Rosenberg Self-Esteem Scale (RSE); Hudson Index of Self-Esteem (ISE); Center for Epidemiologic Studies Depression Scale 10 (CESD-10); Generalized Self-Efficacy Scale (GSES); Emotional/Informational Support subscale of the Medical Outcomes Study Social Support Survey; survey to provide evaluation and feedback
Bohleber et al [103], 2016	Can We Foster a Culture of Peer Support and Promote Mental Health in Adolescence Using a Web-Based App? A Control Group Study	Chatroom	Mobile	Peer, Expert	Unscheduled	Adolescents in Switzerland	1134 (intervention=619; control=515)	Employed: mean age 16.9 (SD 1.73), 50.2% female participants; unemployed: mean age 18.4 (SD 1.96), 40.4% female participants	Quasi experimental study	Yes	The Companion App - Web-based application giving adolescents access to a peer mentoring system and interactive, health-relevant content to strengthen social support and reduce stress	Trier Inventory of Chronic Stress; Satisfaction with social support (Fragebogen zur sozialen Unterstützung, F-Soz-U); qualitative interviews
Lemma and Fonagy [104], 2013	Feasibility study of a psychodynamic online group intervention for depression	Chatroom	Not specified	Expert	Unscheduled	Depression	24 (group intervention=8; self-help intervention=8; control=8)	Anonymous	Feasibility study	Yes	Online Group Dynamic Interpersonal Therapy for depression	Patient Health Questionnaire (PHQ-9); Generalized Anxiety Disorder Scale (GAD-7)

Table S2. Studies using dyadic digital interventions.

Study, year	Title	Multiuser technology	Hardware	Peer or expert	Meeting frequency	Target group	Sample size, N	Demography	Study type	Control group	Intervention—short description	Outcome measures
Schefft et al [105], 2024	Evaluation of the internet-based intervention "Selfapy" in participants with unipolar depression and the impact on quality of life: a randomized, parallel group study	Voice call, chat room	Mobile	Expert	Weekly	Depression	401	Mean age 37.1 (SD 11) | 333 female participants, 68 male participants	Randomized controlled trial	Yes	Selfapy—internet-based intervention for depression	Quality of life The WHOQOL-BREF
Yeo et al [106], 2023	A Digital Peer Support Platform to Translate Online Peer Support for Emerging Adult Mental Well-being: Randomized Controlled Trial	Chat room	Not specified	Peer, expert	Unscheduled	Emerging adulthood (19-25 years)	132 (intervention=82; control=50)	Intervention: mean age 19.44 (SD 1.28 y); control: mean age 19.43 (SD 0.66 y)	Randomized controlled trial	Yes	Acceset—a stand-alone digital peer support platform that uses a digital text-based intervention involving peer disclosure for emerging adult mental well-being	Rosenberg Mattering Scale; General Anxiety Disorder Scale (GAD-7); Multidimensional Scale of Perceived Social Support
Bozkurt and Cesur [107], 2023	The effect of the Ebe Evimde application on the self-efficacy and anxiety levels of mothers: Randomized controlled trial	Videoconference	Mobile, monitor	Expert	Unscheduled	Mothers in the second to fifth postpartum days	120 (intervention=60; control=60)	Intervention: mean age 30.03 (SD 4.05 y); control: mean age 30.83 (SD 3.91 y) | 120 female participants	Randomized controlled trial	Yes	Ebe Evimde (My Home Midwife—web-based software to use in the postpartum period (self-efficacy and anxiety levels	Postpartum Specific Anxiety Scale (pretest and posttest of self-efficacy and anxiety)
Arakawa et al [108], 2023	Effectiveness of mHealth consultation services for preventing postpartum depressive symptoms: a randomized clinical trial	Videoconference, voice call, chat room	Mobile	Expert	Unscheduled	Pregnant women	734 (intervention=365; control=369)	Mean age 32.9 (SD 4.1) | 639 female participants	Randomized controlled trial	Yes	Emotional support related to pregnancy and childcare delivered through the LINE platform	Risk of elevated postpartum depressive symptoms; self-efficacy; loneliness; perceived barriers to health care access; number of clinic visits; ambulance usage
Li et al [109], 2023	Remote arts therapy in collaborative virtual environment: A pilot case study	Virtual world	Head-mounted display	Expert	Weekly	Unspecified—people with higher stress levels	3	2 female participants and 1 male participant	Qualitative study	No	Remote arts therapy in collaborative virtual environment Unity	Perceived Stress Scale; The Warwick-Edinburgh Mental Wellbeing Scale; The System Usability Scale
Crowell et al [10], 2020	Mixed Reality, Full-Body Interactive Experience to Encourage Social Initiation for Autism: Comparison with a Control Non-digital Intervention	Virtual world	Head-mounted display	Peer	Once	High-functioning children with autism	36	Age range 8-12 y | 6 female participants, 30 male participants	Quasi-experimental study	No	Lands of Fog—a Mixed Reality system with full body interactive environment created to foster social and collaborative behaviors in children with autism	Social initiation; HRV EDA; anxiety (STAI); CBCL; collaborative actions
Shorey et al [110], 2019	Evaluation of a Technology-Based Peer-Support Intervention Program for Preventing Postnatal Depression (Part 1): Randomized Controlled Trial	Videoconference, Voice call, chat room	Not specified	Peer	Unscheduled	Mother susceptible to postnatal depression	138 (intervention=69; control=69)	Mean age 32.1 (SD 4.35 y), range 23-43 y | 138 female participants, 0 male participants	Randomized controlled trial	Yes	Technology-based peer-support program for mothers 4 weeks postpartum	Edinburgh Postnatal Depression Scale

Topooco et al [111], 2019	Evaluating the Efficacy of Internet-Delivered Cognitive Behavioral Therapy Blended With Synchronous Chat Sessions to Treat Adolescent Depression: Randomized Controlled Trial	Chat room	Monitor	Expert	Weekly	Adolescents with depression	70 (intervention=35; control=35)	Intervention: mean age 17.5 (SD 1.1 y); control: mean age 17.5 (SD 1.2 y) | 67 female participants	Randomized controlled trial	Yes	ICBT (internet-delivered intervention for adolescent depression	Beck Depression Inventory-II at posttreatment; Mood and Feelings; Questionnaire (MFQ); Beck Anxiety Inventory (BAI); Social Interaction Anxiety Scale (SIAS); General Self-Efficacy scale (GSE); credibility expectancy questionnaire; Working Alliance Inventory (WAI-S); Brunnsviken Brief Quality of Life Scale (BBQ)
Shorey and Ng [112], 2019	Evaluation of a Technology-Based Peer-Support Intervention Program for Preventing Postnatal Depression (Part 2: Qualitative Study	Voice call, chat room	Mobile	Peer	Weekly	Mothers at risk of depression	20 (intervention=10; control=10)	Intervention: mean age 31.5 (SD 4.7 y), range 25-40 y; control: mean age 32.0 (SD 5.0 y), range 25-40 y |39 female participants	Qualitative study	Yes	Technology-based peer-Support Intervention Program for Preventing Postnatal Depression	A qualitative semistructured interview focused on postnatal experience, experience with the program etc.
Depp et al [113], 2010	Mobile Interventions for Severe Mental Illness Design and Preliminary Data From Three Approaches	Chat room	Mobile	Expert	Daily	Bipolar disorder or schizophrenia	10	Mean age 41.0 (SD 13.7 y)	Open trial	No	Mobile interventions for severe mental illness	Qualitative feedback

References

9. Galliers J, Wilson S, Marshall J, Talbot R, Devane N, Booth T, et al. Experiencing EVA Park, a Multi-User Virtual World for People with Aphasia. ACM Trans Access Comput. 2017;10: 1–24. doi:10.1145/3134227

10. Crowell C, Sayis B, Benitez JP, Pares N. Mixed Reality, Full-Body Interactive Experience to Encourage Social Initiation for Autism: Comparison with a Control Nondigital Intervention. Cyberpsychol Behav Soc Netw. 2020;23: 5–9. doi:10.1089/cyber.2019.0115

67. Ronen K, Gewali A, Dachelet K, White E, Jean-Baptiste M, Evans YN, et al. Acceptability and Utility of a Digital Group Intervention to Prevent Perinatal Depression in Youths via Interactive Maternal Group for Information and Emotional Support (IMAGINE): Pilot Cohort Study. JMIR Form Res. 2024;8: e51066. doi:10.2196/51066

68. Pokowitz EL, Prakash N, Planaj D, Oprandi S, Deldin PJ. Mood Lifters for Graduate Students and Young Adults: A Mixed-Methods Investigation into Mechanisms of Change in Online Group Therapy. Behav Sci. 2024;14. doi:10.3390/bs14030252

69. Penwell TE, Smith M, Ortiz SN, Brooks G, Thompson-Brenner H. Traditional versus virtual partial hospital programme for eating disorders: Feasibility and preliminary comparison of effects. Eur Eat Disord Rev. 2024;32: 163–178. doi:10.1002/erv.3031

70. Conroy K, Kehrer SM, Georgiadis C, Hare M, Ringle VM, Shaw AM. Learning from Adolescents and Caregivers to Enhance Acceptability and Engagement Within Virtual Dialectical Behavior Therapy for Adolescents Skills Groups: A Qualitative Study. Child Psychiatry Hum Dev. 2024. doi:10.1007/s10578-023-01641-7

71. Zimmerman M, D'Avanzato C, King BT. Telehealth treatment of patients with major depressive disorder during the COVID-19 pandemic: Comparative safety, patient satisfaction, and effectiveness to prepandemic in-person treatment. J Affect Disord. 2023;323: 624–630. doi:10.1016/j.jad.2022.12.015

72. Xie EB, Freeman M, Penner-Goeke L, Reynolds K, Lebel C, Giesbrecht GF, et al. Building Emotional Awareness and Mental Health (BEAM): an open-pilot and feasibility study of a digital mental health and parenting intervention for mothers of infants. Pilot Feasibility Stud. 2023;9: 27. doi:10.1186/s40814-023-01245-x

73. Wright E, Martinovic J, de Camps Meschino D, Barker LC, Philipp DA, Israel A, et al. A virtual mother-infant postpartum psychotherapy group for mothers with a history of adverse childhood experiences: open-label feasibility study. BMC Psychiatry. 2023;23: 950. doi:10.1186/s12888-023-05444-x

74. Wang C, Darbari I, Tolaymat M, Quezada S, Allen J, Cross RK. Randomized controlled pilot study of feasibility and effectiveness of peer led remote Mindfulness-Based Art Workshops on stress, anxiety, and depression in medical students. Psychol Sch. 2023;60: 1744–1759. doi:10.1002/pits.22809

75. Hunt X, Jivan DC, Naslund JA, Breet E, Bantjes J. South African university students' experiences of online group cognitive behavioural therapy: Implications for delivering digital mental health interventions to young people. Cambridge Prisms: Global Mental Health. 2023;10: e45. doi:10.1017/gmh.2023.39

76. Hollis C, Hall CL, Khan K, Le Novere M, Marston L, Jones R, et al. Online remote behavioural intervention for tics in 9- to 17-year-olds: the ORBIT RCT with embedded process and economic evaluation. Health Technol Assess. 2023;27: 1–120. doi:10.3310/CPMS3211

77. Gerson J, Tawde P, Ghiasian G, Salwen-Deremer JK. Patients' experiences with virtual group gut-directed hypnotherapy: A qualitative study. Front Med. 2023;10: 1066452. doi:10.3389/fmed.2023.1066452

78. Finnerty R, McWeeny S, Trainor L. Online group music therapy: proactive management of undergraduate students' stress and anxiety. Front Psychiatry. 2023;14: 1183311. doi:10.3389/fpsyt.2023.1183311

79. Brownstone LM, Hunsicker MJ, Palazzolo LPA, Dinneen JL, Kelly DA, Stennes J, et al. "Sharing lived experience": Describing a virtual counselor-facilitated LGBTQ+ support group for disordered eating. Psychology of Sexual Orientation and Gender Diversity. 2023. doi:10.1037/sgd0000660

80. Zaccari B, Loftis JM, Haywood T, Hubbard K, Clark J, Kelly UA. Synchronous Telehealth Yoga and Cognitive Processing Group Therapies for Women Veterans with Posttraumatic Stress Disorder: A Multisite Randomized Controlled Trial Adapted for COVID-19. Telemed J E Health. 2022. doi:10.1089/tmj.2021.0612

81. Mi RZ, Kornfield R, Shah DV, Maus A, Gustafson DH. Intraindividual, Dyadic, and Network Communication in a Digital Health Intervention: Distinguishing Message Exposure from Message Production. Health Commun. 2022;37: 397–408. doi:10.1080/10410236.2020.1846273

82. Mendelson D, Thibaudeau É, Sauvé G, Lavigne KM, Bowie CR, Menon M, et al. Remote group therapies for cognitive health in schizophrenia-spectrum disorders: Feasible, acceptable, engaging. Schizophr Res Cogn. 2022;28: 100230. doi:10.1016/j.scog.2021.100230

83. Kruzan KP, Whitlock J, Bazarova NN, Bhandari A, Chapman J. Use of a Mobile Peer Support App Among Young People With Nonsuicidal Self-injury: Small-scale Randomized Controlled Trial. JMIR Formative Research. 2022;6: e26526. doi:10.2196/26526

84. Fell L, Goshe B, Traeger L, Perez G, Iannuzzi D, Park E, et al. Acceptability of A Virtual Mind-Body Group Intervention for Teen Siblings of Children with Autism Spectrum Disorder. J Autism Dev Disord. 2022;52: 5243–5252. doi:10.1007/s10803-022-05500-7

85. El Ayadi AM, Duggal M, Bagga R, Singh P, Kumar V, Ahuja A, et al. A Mobile Education and Social Support Group Intervention for Improving Postpartum Health in Northern India: Development and Usability Study. JMIR Form Res. 2022;6: e34087. doi:10.2196/34087

86. Ali K, Fassnacht DB, Farrer LM, Rieger E, Moessner M, Bauer S, et al. Recruitment, adherence and attrition challenges in internet-based indicated prevention programs for eating disorders: lessons learned from a randomised controlled trial of ProYouth OZ. Journal of Eating Disorders. 2022;10: 1–17. doi:10.1186/s40337-021-00520-7

87. Yeshua-Katz D. The Role of Communication Affordances in Post-Traumatic Stress Disorder Facebook and WhatsApp Support Groups. Int J Environ Res Public Health. 2021;18. doi:10.3390/ijerph18094576

88. Dabit S, Quraishi S, Jordan J, Biagianti B. Improving social functioning in people with schizophrenia-spectrum disorders via mobile experimental interventions: Results from the CLIMB pilot trial. Schizophr Res Cogn. 2021;26: 100211. doi:10.1016/j.scog.2021.100211

89. Craig SL, Leung VWY, Pascoe R, Pang N, Iacono G, Austin A, et al. AFFIRM Online: Utilising an Affirmative Cognitive-Behavioural Digital Intervention to Improve Mental Health, Access, and Engagement among LGBTQA+ Youth and Young Adults. Int J Environ Res Public Health. 2021;18. doi:10.3390/ijerph18041541

90. Craig SL, Iacono G, Pascoe R, Austin A. Adapting Clinical Skills to Telehealth: Applications of Affirmative Cognitive-Behavioral Therapy with LGBTQ+ Youth. Clin Soc Work J. 2021;49: 471–483. doi:10.1007/s10615-021-00796-x

91. Abedishargh N, Farani AR, Gharraee B, Farahani H. Effectiveness of Internet-based Cognitive Behavioral Therapy in Weight Loss, Stress, Anxiety, and Depression via Virtual Group Therapy. Iranian Journal of Psychiatry and Behavioral Sciences. 2021;15. doi:10.5812/ijpbs.113096

92. Tsai H-H, Cheng C-Y, Shieh W-Y, Chang Y-C. Effects of a smartphone-based videoconferencing program for older nursing home residents on depression, loneliness, and quality of life: a quasi-experimental study. BMC Geriatr. 2020;20: 27. doi:10.1186/s12877-020-1426-2

93. Strand M, Eng LS, Gammon D. Combining online and offline peer support groups in community mental health care settings: a qualitative study of service users' experiences. Int J Ment Health Syst. 2020;14: 1–12. doi:10.1186/s13033-020-00370-x

94. Robinson-Whelen S, Hughes RB, Taylor HB, Markley R, Vega JC, Nosek TM, et al. Promoting psychological health in women with SCI: Development of an online self-esteem intervention. Disabil Health J. 2020;13: 100867. doi:10.1016/j.dhjo.2019.100867

95. Vogel EA, Thrul J, Humfleet GL, Delucchi KL, Ramo DE. Smoking cessation intervention trial outcomes for sexual and gender minority young adults. Health Psychol. 2019;38: 12–20. doi:10.1037/hea0000698

96. Tait RJ, Paz Castro R, Kirkman JJL, Moore JC, Schaub MP. A Digital Intervention Addressing Alcohol Use Problems (the "Daybreak" Program): Quasi-Experimental Randomized Controlled Trial. J Med Internet Res. 2019;21: e14967. doi:10.2196/14967

97. Sawyer A, Kaim A, Le H-N, McDonald D, Mittinty M, Lynch J, et al. The Effectiveness of an App-Based Nurse-Moderated Program for New Mothers With Depression and Parenting Problems (eMums Plus): Pragmatic Randomized Controlled Trial. J Med Internet Res. 2019;21: e13689. doi:10.2196/13689

98. Campbell A, Ridout B, Amon K, Navarro P, Collyer B, Dalgleish J. A Customized Social Network Platform (Kids Helpline Circles) for Delivering Group Counseling to Young People Experiencing Family Discord That Impacts Their Well-Being: Exploratory Study. J Med Internet Res. 2019;21: e16176. doi:10.2196/16176

99. Granado-Font E, Ferré-Grau C, Rey-Reñones C, Pons-Vigués M, Pujol Ribera E, Berenguera A, et al. Coping Strategies and Social Support in a Mobile Phone Chat App Designed to Support Smoking Cessation: Qualitative Analysis. JMIR Mhealth Uhealth. 2018;6: e11071. doi:10.2196/11071

100. Knowles LM, Stelzer E-M, Jovel KS, O'Connor M-F. A pilot study of virtual support for grief: Feasibility, acceptability, and preliminary outcomes. Comput Human Behav. 2017;73: 650–658. doi:10.1016/j.chb.2017.04.005

101. Carolan S, Harris PR, Greenwood K, Cavanagh K. Increasing engagement with an occupational digital stress management program through the use of an online facilitated discussion group: Results of a pilot randomised controlled trial. Internet Interv. 2017;10: 1–11. doi:10.1016/j.invent.2017.08.001

102. Nosek MA, Robinson-Whelen S, Hughes RB, Nosek TM. An Internet-based virtual reality intervention for enhancing self-esteem in women with disabilities: Results of a feasibility study. Rehabil Psychol. 2016;61: 358–370. doi:10.1037/rep0000107

103. Bohleber L, Crameri A, Eich-Stierli B, Telesko R, von Wyl A. Can We Foster a Culture of Peer Support and Promote Mental Health in Adolescence Using a Web-Based App? A Control Group Study. JMIR Ment Health. 2016;3: e45. doi:10.2196/mental.5597

104. Lemma A, Fonagy P. Feasibility study of a psychodynamic online group intervention for depression. Psychoanal Psychol. 2013;30: 367–380. doi:10.1037/a0033239

105. Schefft C, Krämer R, Haaf R, Jedeck D, Schumacher A, Köhler S. Evaluation of the internet-based intervention "Selfapy" in participants with unipolar depression and the impact on quality of life: a randomized, parallel group study. Qual Life Res. 2024;33: 1275–1286. doi:10.1007/s11136-024-03606-2

106. Yeo G, Loo G, Oon M, Pang R, Ho D. A Digital Peer Support Platform to Translate Online Peer Support for Emerging Adult Mental Well-being: Randomized Controlled Trial. JMIR Ment Health. 2023;10: e43956. doi:10.2196/43956

107. Bozkurt MA, Cesur B. The effect of the Ebe Evimde application on the self-efficacy and anxiety levels of mothers: Randomized controlled trial. Digit Health. 2023;9: 20552076231169840. doi:10.1177/20552076231169840

108. Arakawa Y, Haseda M, Inoue K, Nishioka D, Kino S, Nishi D, et al. Effectiveness of mHealth consultation services for preventing postpartum depressive symptoms: a randomized clinical trial. BMC Med. 2023;21: 1–12. doi:10.1186/s12916-023-02918-3

109. Li C, Yip PY. Remote arts therapy in collaborative virtual environment: A pilot case study. Front Virtual Real. 2023;4. doi:10.3389/frvir.2023.1059278

110. Shorey S, Chee CYI, Ng ED, Lau Y, Dennis C-L, Chan YH. Evaluation of a Technology-Based Peer-Support Intervention Program for Preventing Postnatal Depression (Part 1): Randomized Controlled Trial. J Med Internet Res. 2019;21: e12410. doi:10.2196/12410

111. Topooco N, Byléhn S, Dahlström Nysäter E, Holmlund J, Lindegaard J, Johansson S, et al. Evaluating the Efficacy of Internet-Delivered Cognitive Behavioral Therapy Blended With Synchronous Chat Sessions to Treat Adolescent Depression: Randomized Controlled Trial. J Med Internet Res. 2019;21: e13393. doi:10.2196/13393

112. Shorey S, Ng ED. Evaluation of a Technology-Based Peer-Support Intervention Program for Preventing Postnatal Depression (Part 2): Qualitative Study. J Med Internet Res. 2019;21: e12915. doi:10.2196/12915

113. Depp CA, Mausbach B, Granholm E, Cardenas V, Ben-Zeev D, Patterson TL, et al. Mobile interventions for severe mental illness: design and preliminary data from three approaches. J Nerv Ment Dis. 2010;198: 715–721. doi:10.1097/NMD.0b013e3181f49ea3
